# Translating a Group-Based Dyadic Intervention for Early-Stage People and Their Care Partners for Spanish Speakers

**DOI:** 10.1093/geroni/igaa057.2136

**Published:** 2020-12-16

**Authors:** David Coon, Marielysse Cortes, Abigail Gomez, Berta Carbajal, Maria Gonzalez, Lourdes Cordova, Zenya Weatherall

**Affiliations:** Arizona State University, Phoenix, Arizona, United States

## Abstract

This presentation focuses on the development of and recruitment for a Spanish language version of EPIC, a group-based dyadic intervention for people with early-stage dementia (EPs) and their care partners (CPs). EPIC involves activities for the entire group together, separate EP or CP groups, and one individualized session for each dyad. EPIC builds on components of SHARE combined with strategies from other psychoeducational interventions (e.g., skills-training, care values clarification, care planning). Findings from 6 focus groups with Latino participants were used to help with cultural responsiveness such as fluent Spanish/bilingual staff, testimonials from Latino families affected by dementia, and minimization of “threatening” language for memory loss. Participants also provided suggestions for recruitment for Spanish speakers such as engaging promotores, using social media across generations, recruiting through Spanish language health classes, and recognizing the impact of the political environment. The presentation will review both recruitment successes and challenges using these approaches.

